# Analysis of the Accuracy of the Modified CT Severity Index in Predicting Clinical Outcomes in Acute Pancreatitis: A Cross-Sectional Study

**DOI:** 10.7759/cureus.56123

**Published:** 2024-03-13

**Authors:** Mathew John Mathai, Vijay Sai Reddy M, Varun Shetty

**Affiliations:** 1 Department of General Surgery, Dr. D. Y. Patil Medical College, Hospital & Research Centre, Dr. D. Y. Patil Vidyapeeth, Pune, IND

**Keywords:** acute pancreatitis, modified ct severity index, prognosis, ranson score, computed tomography, alcohol-induced pancreatitis, pancreas

## Abstract

Objective: To evaluate the accuracy of the modified CT severity index (MCTSI) in predicting the severity of acute pancreatitis and to prognosticate the clinical outcomes.

Methods and materials: The study was conducted at a tertiary health center between January 2021 and June 2023. A total of 150 consecutive patients with clinical/laboratory features suggestive of acute pancreatitis were included in the study and underwent a contrast-enhanced CT scan within 24 hours of admission. Based on their MCTSI score, these patients had conservative or surgical/endoscopic treatment. Clinical outcomes were assessed in terms of recovery, development of complications, or death. The receiver operating characteristic curve and descriptive statistics were computed to determine the sensitivity and specificity. The data were analyzed using SPSS version 16 software (SPSS Inc., Chicago, IL), and an attempt was made to evaluate the accuracy of MCTSI in predicting these clinical outcomes.

Results: The mean age of patients in our study was 49.21 ± 11.02 years. Out of the 150 included patients, 103 were men and 47 were women. Compared to 11.68% of severe acute pancreatitis patients who died, 88.32% recovered. The area under the curve was determined as 0.865, based on which the MCTSI score predicted acute pancreatitis clinical outcome with 64% sensitivity and 92% specificity. The MCTSI demonstrated value in predicting clinical outcomes with a p-value of 0.043 ± 0.012 (p < 0.05) in the recovered patients while p = 0.032 ± 0.012 for patients who succumbed. The p-value for MCTSI in predicting complications was p = 0.0012 ± 0.0008 (p < 0.05).

Conclusion: Our study was able to demonstrate the high level of accuracy of the MCTSI score in predicting complications and clinical outcomes, especially in patients with severe acute pancreatitis. The MCTSI serves as a valuable asset in the preliminary evaluation of acute pancreatitis, thereby facilitating appropriate management.

## Introduction

Acute pancreatitis has long been recognized for its diverse range of manifestations, spanning from mild, nonspecific symptoms to the development of organ failure. Over the course of time, numerous investigations have been utilized to evaluate the extent and customize the management. Nevertheless, it is important to note that no individual investigation possesses the capacity to comprehensively evaluate the disease, resulting in the persistence of substantial morbidity and mortality in relation to this condition [1].

At present, contrast-enhanced computed tomography (CECT) is the preferred imaging modality in acute pancreatitis as it not only helps in assessing the extent of inflammation but also helps in determining the severity and associated complications. Balthazar and colleagues introduced a grading system (CT severity index - CTSI) for acute pancreatitis that involved assessing the size, shape, and density of the pancreas, as well as the presence of peripancreatic abnormalities, to predict the severity of the disease [2]. However, the original CTSI did not take into account the extra-pancreatic complications, which significantly affect the prognosis.

Considering these constraints, Mortele et al. put forth a modified and streamlined CT scoring system (modified CT severity index, MCTSI) that offers simplicity in computation and replication while exhibiting stronger associations with patient outcome indicators such as infection rates, organ dysfunction, the need for percutaneous intervention or surgery, length of hospital stays, and mortality compared to the CTSI [3,4].

The objective of this study was to investigate the accuracy of the MCTSI in predicting the clinical outcomes of individuals diagnosed with acute pancreatitis.

## Materials and methods

This prospective cross-sectional research was conducted between 2021 and 2023 at a tertiary care health center. Included in the investigation were 150 consenting study participants with acute pancreatitis (by convenience sampling). Institutional ethics clearance was obtained prior to beginning the study (I.E.S.C./170/2021). Informed consent was obtained from all participants prior to the initiation of the study. All referred patients presenting with clinical/laboratory features of acute pancreatitis (as mentioned below) were included in the study.

The diagnostic criteria employed for inclusion in the study were based on the presence of clinical/biochemical features of acute pancreatitis. These included clinical (acute abdominal pain and tenderness consistent with pancreatitis) and biochemical (serum amylase/lipase three times the normal) parameters.

Patients with a known history of allergy to iodinated contrast agents and deranged renal function test (serum creatinine > 1.5 mg/dl after rehydration) were excluded from the study. In addition, pregnant patients and those below 18 years of age were also excluded. All included patients underwent a CECT of the abdomen and pelvis (48 hours after admission), and an MCTSI score was assigned to each participant (Table 1) [5].

**Table 1:** Modified CT severity index (MCTSI) score Mild acute pancreatitis: 0-2. Moderate acute pancreatitis: 2-4. Severe acute pancreatitis: 6 and above.

MCTSI score	Pancreatic condition
Pancreatic inflammation	
0	Normal pancreas
2	Intrinsic pancreatic abnormality with or without inflammation
4	Pancreatic or peri-pancreatic fluid collection or peri-pancreatic fat necrosis
Pancreatic necrosis	
0	None
2	30% or less
4	More than 30%
Extra-pancreatic complications	
2	One or more of the following: pleural effusion, ascites, vascular complications, parenchymal complications, and/or gastrointestinal involvement

All the included participants were scanned thoroughly by means of detailed clinical examination and biochemical evaluation (including arterial blood gas, complete blood count, serum electrolytes, renal and liver function tests, and coagulation profile), and the findings were logged in dedicated proformas created for this research. In addition, MCTSI scores were assigned to each participant based on the CECT of the abdomen and pelvis findings.

Patients with an MCTSI of less than 6 were treated primarily with a conservative approach. Adequate fluid resuscitation was achieved according to body weight. A mean arterial pressure (MAP) greater than 60 mmHg was maintained during resuscitation. Urine output of a minimum of 1 ml/kg body wt./hour was maintained. All the patients were kept nil by mouth (NBM) with a nasogastric tube for about two to three days till the patients settled down followed by a liquid and soft diet. Adequate analgesia and prophylactic antibiotics in the management of local as well as systemic complications of acute pancreatitis were administered. Somatostatin analogs were also administered as a part of the conservative approach to all the included patients (except in patients presenting with severe hypotension).

Patients with MCTSI scores of 6 and above were initially managed with the conservative approach as mentioned above. Patients with an MCTSI score of more than 8 and developing possible complications (local/systemic) were then subjected to appropriate endoscopic/operative intervention (endoscopic retrograde cholangiopancreatography with or without pancreatic duct/common bile duct stenting, necrosectomy - open/closed, drainage of pancreatic abscess, etc.).

Patient’s MCTSI scores were then correlated with the development of possible complications, as mentioned above, using chi-square of statistical significance to establish the accuracy of MCTSI in predicting clinical outcomes. Clinical outcome assessment was based on the duration of hospital stay, mortality rates, and possible recurrence (followed up after six months, one year, and two years wherein detailed physical examination and CECT were carried out to look for recurrence. These recorded clinical outcomes were compared with the MCTSI scores at admission, to evaluate the ability of MCTSI to predict clinical outcomes.

The receiver operating characteristic (ROC) curve and descriptive statistics were computed to determine the sensitivity and specificity based on the area under the curve. After completion of data collection, data analysis was achieved using SPSS version 16 software (SPSS Inc., Chicago, IL), and the correlations sought after were achieved using the chi-square test of significance.

## Results

The age group with the highest incidence of acute pancreatitis in our study was the fourth decade. The mean age of study participants was 48.21 ± 11.02 (mean ± standard deviation). Of the 150 patients, 103 were males and 47 were females. The observed sex ratio was 2.32:1, indicating a higher proportion of males (Tables 2, 3).

**Table 2:** Demographic parameters of the participants (age)

Age (years)	N (%)
21-30	8 (12%)
31-40	24 (16%)
41-50	50 (33.33%)
51-60	32 (21.33%)
61-70	29 (19.33%)
>71	7 (4.66%)
Total	150

**Table 3:** Demographic parameters (gender)

Gender	Number (n)
Male	103 (68.66%)
Female	47 (31.33%)
Total	150

The majority of the participants were employed as laborers, i.e., 43.33% (n = 65), with housewives comprising the second largest occupational group, i.e., 25.67% (n = 39). According to the modified Kuppuswamy scale, the majority of the study participants fall within the lower and lower middle socioeconomic strata. Alcoholism was shown to be the predominant etiology in 61.5% (n = 92), followed by gallstones-induced pancreatitis (29%, n = 43) as the second leading etiology. The majority of our study participants, i.e., 68.5% (n = 103) did not exhibit any comorbidities, although 18.91% (n = 28) presented with diabetes mellitus, and 11.5% (n = 17) had hypertriglyceridemia, both of which are considered contributory factors. A total of 85% (n = 128) of the patients had elevated levels of serum amylase, more than three times the ceiling of 240 IU/L, and also demonstrated elevated levels of serum lipase, surpassing four times the standard limit of 320 IU/L.

The average duration of hospitalization in our research was found to be 15.23 ± 4.66 days. The average MCTSI score in our sample was 6.4 ± 2.81 (mean ± standard deviation). Among the cohort of 150 patients, a majority of 97 individuals were subjected to conservative management. Subsequently, during the follow-up period, 15 patients underwent cholecystectomy, 14 patients underwent percutaneous pancreatic abscess drainage, 18 patients underwent laparoscopic necrosectomy, and six patients underwent open necrosectomy.

The MTSCI score demonstrated a statistically significant association with the development of complications both local as well as systemic (Table 4). In a sample size of 150 people who were subjected to assessment, a total of 88 patients experienced local/systemic complications ranging from pseudocyst formation and pancreatic necrosis to more fatal ones like multi-organ failure. The majority of the complications documented developed in cases with an MCTSI score of 8-10 (81/150) whereas the 0-2 and 4-6 score groups reported one and six patients with complications, respectively, thereby depicting a positive correlation with the MCTSI score.

**Table 4:** Correlation of MCTSI score at presentation with complications (local/systemic) MCTSI: modified CT severity index.

MCTSI score	Complications		Total	P-value = 0.0012 ± 0.0004 (p < 0.05)
	Yes	No		
0-2	1 (5.88%)	16 (94.11%)	17	
4-6	6 (12.5%)	42 (87.5%)	48	
8-10	81 (95.29%)	4 (47.05%)	85	
Total	88 (58.66%)	62 (41.33%)	150	

In our study, the area under the curve was determined to be 0.865, based on which the sensitivity and specificity of 64% and 92% were derived. The negative and positive predictive values for MCTSI in this study were 60% and 90%, respectively. In contrast to the mortality rate of 11.68% (n = 9) observed among patients diagnosed with severe acute pancreatitis (MCTSI score: 8-10), a significant majority of patients (88.31%, n = 68) with MCTSI scores less than 8 demonstrated recovery. The p-value obtained from the MCTSI analysis for predicting clinical outcomes (in the recovered group) was found to be p = 0.043 ± 0.012 (p < 0.05). The p-value obtained from the MCTSI analysis for predicting clinical outcomes (in the death group) was found to be p = 0.032 ± 0.012 (p < 0.05), suggesting statistically significant findings (Table 5).

**Table 5:** Correlation of MCTSI score at presentation with clinical outcome MCTSI: modified CT severity index.

MCTSI score	Clinical outcome		Total	p = 0.043 ± 0.012 (p < 0.05) in terms of recovered patients and p = 0.032 ± 0.012 (p < 0.05) for those who succumbed.
	Recovered	Death		
0-2	24 (96%)	1 (4%)	25	
4-6	47 (97.91%)	1 (2.08%)	48	
8-10	68 (88.31%)	9 (11.68%)	77	
Total	139 (92.66)	11 (73.33%)	150	

## Discussion

Acute pancreatitis is an often-observed medical disease with varied presentations and clinical outcomes depending on the clinical severity at presentation. The early and prompt recognition of severe cases allows the treating physicians to provide more aggressive care in contrast to more conservative measures in less severe cases, thereby allowing for better resource allocation. To establish a diagnosis of acute pancreatitis, it is necessary to conduct a full evaluation encompassing a detailed medical history, a thorough physical examination, and the performance of laboratory tests. The potential requirement for radiological verification may arise. The present investigation examines the clinical symptoms associated with acute pancreatitis [5-8].

The relevant investigations were completed and managed efficiently, taking into consideration the origin and severity of acute pancreatitis. In line with the studies conducted by Nandu et al. and Pupelis et al., the mean age of onset in our investigation was recorded as 49.21 years, as seen in Table 6 [9,10]. The observed phenomenon can be attributed to the prominence of alcohol as the principal causal factor in our research, which tends to present itself at a younger age.

**Table 6:** Comparison of mean age with other studies

Studies	Mean age in years
Kashid, 2006 [8]	35
Nandu et al., 2016 [9]	44.8
Pupelis et al., 2008 [10]	36.2
Choudhuri, 2006 [11]	56.3

In our study, the proportion of male patients was around 68.66% (n = 103), resulting in a male-to-female ratio of 2.32 to 1. Previous studies also exhibited a greater representation of male participants, including those conducted by Pupelis et al. [10] and Koizumi et al. [12] (Table 7). The primary causal factor for this phenomenon can be attributed to the consumption of alcohol (n = 92), followed closely by gallstones-induced pancreatitis (n = 43). Among the patient population under consideration, it was observed that 16.66% (n = 25) exhibited mild acute pancreatitis, while 32% (n = 48) displayed symptoms indicative of moderately severe acute pancreatitis. The remaining 51.33% (n = 77) of patients were diagnosed with severe acute pancreatitis. This finding shared similarities with the study by Lee et al. [5].

**Table 7:** Comparison of gender statistics with other similar studies

Studies	Males	Females
Pupelis et al., 2008 [10]	90	38
Koizumi et al., 2006 [12]	100	60

In our study, the MCTSI score had a sensitivity of 64% and a specificity of 98% in predicting clinical outcomes. The negative and positive predictive values for MCTSI in this study were 60% and 90%, respectively. These findings were along the same lines as a study conducted by Jauregui et al., stating that for the MCTSI to detect severe pancreatitis, sensitivity, specificity, and positive predictive values were 61%, 90%, and 91%, respectively [13-18]. Thus MCTSI served as an accurate instrument in our study to predict clinical outcomes in patients with severe acute pancreatitis.

However, it is important to note that the significance of the findings may be restricted due to the small study population and the narrow range of factors that were included in the analysis. The p-value for the MCTSI in its ability to predict clinical outcomes was found to be p = 0.043 ± 0.012 (p < 0.05) in terms of recovered patients and p = 0.032 ± 0.012 (p < 0.05) for those who succumbed, indicating statistical significance.

The p-value for the MCTSI in its ability to predict complications was found to be p = 0.0012 ± 0.0008 (p < 0.05), indicating statistical significance. The observed consequences encompassed a spectrum of manifestations, spanning from mild hypotension and temporary organ failure to the more severe end of the spectrum, with septic shock and multiple organ failure [19-21]. The high specificity and positive predictive values in cases with severe acute pancreatitis (MCTSI score > 8-10) indicated its significant role in these cases as compared to mild and moderate cases of pancreatitis. Additionally, it can aid in estimating potential clinical outcomes and complications, enabling surgeons to deliver personalized treatment to patients and subsequently reducing both morbidity and mortality rates.

The study was subject to certain limitations, namely, a very small sample size, a lack of measures taken to avoid bias, and a limited duration of follow-up. Nevertheless, our intention is to further our research by incorporating a larger study cohort, thus yielding more comprehensive data that can contribute to the expanding body of information regarding acute pancreatitis.

## Conclusions

Based on the findings of our study, MCTSI is an accurate and valuable tool in the evaluation and initial assessment of acute pancreatitis. The study showed a high specificity and positive predictive values indicating a high accuracy, especially in severe acute pancreatitis.

The present study demonstrates a statistically significant association between the MCTSI and clinical outcomes, suggesting its potential utility in accurately diagnosing potential complications and revealing its high sensitivity in the routine evaluation of acute pancreatitis.
